# Long-Chain Omega-3 Polyunsaturated Fatty Acids Have Developmental Effects on the Crop Pest, the Cabbage White Butterfly *Pieris rapae*

**DOI:** 10.1371/journal.pone.0152264

**Published:** 2016-03-24

**Authors:** Stefanie M. Hixson, Kruti Shukla, Lesley G. Campbell, Rebecca H. Hallett, Sandy M. Smith, Laurence Packer, Michael T. Arts

**Affiliations:** 1 Department of Chemistry and Biology, Ryerson University, Toronto, Ontario, Canada; 2 School of Environmental Sciences, University of Guelph, Guelph, Ontario, Canada; 3 Department of Forestry, University of Toronto, Toronto, Ontario, Canada; 4 Department of Biology, York University, Toronto, Ontario, Canada; CINVESTAV-IPN, MEXICO

## Abstract

Nutritional enhancement of crops using genetic engineering can potentially affect herbivorous pests. Recently, oilseed crops have been genetically engineered to produce the long-chain omega-3 polyunsaturated fatty acids, eicosapentaenoic acid (EPA) and docosahexaenoic acid (DHA) at levels similar to that found in fish oil; to provide a more sustainable source of these compounds than is currently available from wild fish capture. We examined some of the growth and development impacts of adding EPA and DHA to an artificial diet of *Pieris rapae*, a common pest of Brassicaceae plants. We replaced 1% canola oil with EPA: DHA (11:7 ratio) in larval diets, and examined morphological traits and growth of larvae and ensuing adults across 5 dietary treatments. Diets containing increasing amounts of EPA and DHA did not affect developmental phenology, larval or pupal weight, food consumption, nor larval mortality. However, the addition of EPA and DHA in larval diets resulted in progressively heavier adults (F _4, 108_ = 6.78; p = 0.011), with smaller wings (p < 0.05) and a higher frequency of wing deformities (R = 0.988; p = 0.001). We conclude that the presence of EPA and DHA in diets of larval *P*. *rapae* may alter adult mass and wing morphology; therefore, further research on the environmental impacts of EPA and DHA production on terrestrial biota is advisable.

## Introduction

Fatty acids (FA) have multifaceted roles in metabolic energy storage, cell membrane structure, temperature acclimation, cell signalling, cognition, vision, and the immune system in both aquatic and terrestrial organisms [1–3]. The 18-carbon omega-3 (n-3) and omega-6 (n-6) polyunsaturated fatty acids (PUFA), alpha-linolenic acid (ALA; 18:3n-3) and linoleic acid (LNA; 18:2n-6), respectively, are essential to most animals (including insects; 2), as they cannot be synthesized *de novo*, and therefore must be obtained from the diet. The long-chain n-3 polyunsaturated fatty acids (LC-PUFA), eicosapentaenoic acid (EPA; 20:5n-3) and docosahexaenoic acid (DHA; 22:6n-3) are involved with key physiological functions in both aquatic invertebrates and vertebrates [3], yet most aquatic invertebrates and vertebrates cannot synthesize these two LC-PUFA at rates sufficient to maintain an optimal state [4]. EPA and DHA mainly originate in aquatic primary producers (algae) and are generally retained up the food chain via successive consumption by increasingly higher trophic level organisms [5]. EPA and DHA are novel to the terrestrial environment, as they are, for the most part, not produced by terrestrial plants and are not transferred through terrestrial food webs, unless consumed from aquatic resources or produced endogenously [6, 7].

Although humans (and other vertebrates) have relied on aquatic resources for most of their EPA and DHA, many wild fish stocks are now at or beyond exploitable limits and cannot further increase to satisfy the growing human demand for seafood [8]. While aquaculture is currently filling this gap between supply and demand, sufficient levels of EPA and DHA in farmed fish are presently only acquired through the use of fish oil supplied in the feed and which, currently, is derived mainly from wild fish capture [9, 10]. Therefore, growth of the finfish aquaculture industry is increasingly limited by access to sustainable sources of EPA and DHA [11, 12]. Because of the increasing demand for EPA and DHA, science and industry have recognized a need to find alternate methods to produce these essential fatty acids sustainably, i.e. without relying on over-exploited wild fisheries [11–13]. As a solution to this potential global shortage, oilseed crops (e.g. canola, *Brassica napus* L., and camelina, *Camelina sativa* L., Crantz) have been genetically engineered, through insertion of genes derived from algae, to produce EPA and DHA at levels similar to that of fish oil [14–16]. The oil produced from these crops can replace traditional, wild-sourced, fish oils used in the production of aquaculture and livestock feeds, pharmaceuticals, and functional foods [12, 13]. Because EPA and DHA are largely novel FA at the level of terrestrial primary producers and terrestrial insects [11, 12], and have not yet entered the agroecosystem, the effects of their consumption on terrestrial insect growth and development are unknown, and have not been the subject of any scientific study to date.

Instead, studies of the nutritional consequences of consuming EPA and DHA have focused on aquatic invertebrates, where these compounds have been shown to exert strong positive effects on growth and reproduction [3]. Additionally, the FA composition of terrestrial insects is generally characterized by the absence of 20-carbon (and greater) n-3 PUFA, i.e., lack of EPA and DHA. In contrast, arachidonic acid (ARA; 20:4n-6), is common in the terrestrial ecosystem, and is known to play a key role in cell membrane structure, reproduction, and immune function in terrestrial insects [1, 17]. However, the effect of EPA and DHA on the growth, development, and/or reproduction of terrestrial insects is unknown.

Genetically engineered oilseed crops have reached a mature stage of development at a time when demand (and costs) for fish oils, from aquaculture and nutraceutical industries, continues to rise. Simultaneously, it is clear that insect pests, which may be affected by the introduction of EPA and DHA, represent an ongoing challenge to global food security [18]. Consequently, the purpose of our experiment was to preliminarily assess whether consumption of EPA and DHA would alter the growth and/or development of an insect pest in a carefully-controlled laboratory setting. We tested these objectives using a common crop pest, the cabbage white butterfly (*Pieris rapae* L.; Lepidoptera: Pieridae), which consumes leaf tissue and causes significant and widespread economic damage to plants in the family Brassicaceae, which include canola and camelina [19]. Based on the known responses of aquatic invertebrates to dietary EPA and DHA, we hypothesized that EPA and DHA, provided in artificial diets of *P*. *rapae* larvae, might similarly affect development resulting in changes in growth rate, body size or other characteristics. Therefore, our primary objective was to preliminarily assess the potential effects of dietary EPA and DHA on the growth and development of *P*. *rapae*, in order to better understand the possible impacts of commercial production of novel oilseed crops on insect pests.

## Methods

### Study organism

*Pieris rapae* larvae feed on a narrow range of hosts, mainly in the family Brassicaceae [19]. The geographic distribution of *P*. *rapae* extends across Europe, North Africa, and Asia. It has been introduced to North America, Australia, and New Zealand, and was brought to Canada in 1860 [20]. The larval stage is an economic pest of Brassica crops, including canola [19]. The larvae appear to prefer cultivated plants over wild plants [21]. The life cycle includes 5 larval instars, followed by pupation and maturation as an adult butterfly [22]. At 21°C, the time from egg to adult is ~1 month [22]. Adults live for ~3 weeks [21].

### Experimental design

Eggs were obtained from the Carolina Biological Supply Company (Burlington, North Carolina) and were reared according to Webb and Shelton [22] and the instructions provided by the supplier. Briefly, eggs were kept at 25 ± 1°C and 30% relative humidity, under diapause-inducing conditions (12 h light: 12 h dark). Each egg strip (~20 eggs per strip) was placed, egg-side down, in a plastic translucent Dixie cup (163 mL) with ~20 mL of diet in the bottom of the cup. The cups were sealed with lids, each with one hole for ventilation, and one hole plugged with a Q-tip dipped in water to maintain constant humidity in the container. The eggs hatched within 24 h of arrival and the larvae remained in the containers with control diet for an additional 3 days to ensure robustness of the larvae prior to transfer. On the fourth day after hatch, the larvae were transferred individually (n = 150) into plastic translucent housing cups (60 mL) with experimental diets (~5 mL of diet each). The experimental units were arranged as a randomized block design in order to control for potential sources of environmental variability in the room (e.g. light, noise, vibration, etc.), with 25 units per block (6 blocks total), with 5 replicates per treatment in each block, and 30 replicates per treatment total.

### Experimental diets

Larvae were provided 1 of 5 dietary treatments, slightly modified from Webb and Shelton’s [22] formulation which we calculated to contain ~10% fat (derived entirely from wheat germ). In the control diet, canola oil was added at 1% of the diet in addition to the estimated 10% fat supplied from wheat germ. In the experimental diets, EPA and DHA incrementally replaced canola oil in the following ratios: 0:1 (hereafter referred to as control), 0.25:0.75 (lowest), 0.5:0.5 (low), 0.75:0.25 (medium), and 1:0 (high). We maintained an EPA: DHA ratio of 11:7 in all diets, as this was representative of the ratio found in genetically engineered camelina oil [16]. However, the total lipid amount in the diet was estimated based on the published formulation for this species [22], consistent with the proven and widely used method of rearing this species. All dietary ingredients were obtained from Bio-Serv, Inc. (Flemington, New Jersey), except for EPA and DHA, which was isolated (both at 99% purity) from algae (Matreya LLC, Pleasant Gap, Pennsylvania).

### Growth and morphology measurements

On the 11^th^ day after hatch, larvae were weighed and placed on fresh diet. On the 18^th^ day after hatch, larvae were given fresh diet (0.01 g) which was weighed daily until pupation. Larvae were weighed at the 5^th^ instar, prior to pupation. Pupae were weighed and transferred to a container without diet. After the emergence of each individual, the individual butterflies were kept in their containers up to 48 h to ensure full emergence and wing expansion. Fully emerged butterflies were sacrificed by placing them in a -20°C freezer for several minutes until movement ceased and mortality was presumed. They were then weighed (wet weight) and pinned (the wings were flattened and gently pressed) to an insect board to measure wing span (forewing and hindwing; between wing tips), total wing length (forewing base to apex), forewing and hindwing length (partial length) and body length (tip of head to tip of abdomen), according to Chai and Srygley [23].

### Lipid and fatty acid analysis

After measurements were taken, butterflies were placed individually into 2.5 mL cryogenic vials and frozen at -80°C; after which they were freeze-dried. Whole, freeze-dried, butterflies were individually ground to a fine powder, in liquid nitrogen, using a mortar and pestle, and the ensuing powder was weighed to the nearest microgram. Total lipid was extracted using a modified Folch et al. [24] method, as in [25]. In brief, each sample was extracted three times, using 2 mL of chloroform/methanol (2:1; v/v) and then pooled (total 6 mL). Polar impurities were removed by adding 1.6 mL NaCl solution (0.9% w/v); this layer was discarded following centrifugation. The resulting lipid-containing solvent was concentrated to 2 mL and 2 aliquots (100 μL each) were removed and evaporated to dryness to determine total lipid through gravimetric analysis. The lipid extract was then prepared for gas chromatography by derivatizing FA (including EPA and DHA) to FA methyl esters (FAME) using sulfuric acid as the catalyst [26]. Fatty acid methyl esters were extracted twice using hexane: diethyl ether (1:1; v/v), then dried under a gentle stream of nitrogen. The dry FAME extract was re-dissolved in hexane and individual FAME were separated using a gas chromatograph (GC) (Shimadzu-2010 Plus, Nakagyo-ku, Kyoto, Japan) equipped with an SP-2560 column (Sigma-Aldrich, St. Louis, Missouri). All solvents used in the extraction and FAME derivatization procedures were of high purity HPLC grade (>99%). FAME in samples were identified by comparison of their retention times with a known standard (GLC-463 reference standard; Nu-chek Prep, Inc., Waterville, Minnesota) and quantified with a 5-point calibration curve using this same standard. A known concentration of 5 alpha-cholestane (C8003, Sigma-Aldrich, St. Louis, Missouri) was added to each sample prior to extraction to act as a surrogate internal standard to estimate extraction and instrument recovery efficiency.

### Statistical analysis

The experimental units (n = 30 per treatment) were arranged in 6 blocks (n = 25 individuals per block) following a randomized complete block design (Minitab 16 Statistical Software). Using ANOVA, we tested the hypotheses that dietary treatment (fixed factor) had an effect on the response variables, development (time to pupation, emergence, and pupation to emergence) and morphology (larval, pupal, and adult mass, and wing span and length). This design was also used to test the hypothesis that dietary treatment (fixed factor) affected the fatty acid amounts in the whole butterfly.

Linear regression and correlation analyses were used to test the direct effect of dietary EPA and DHA amounts (independent variables) on specific measurements (dependent variables) (SigmaPlot 11.0, Systat Software, Inc.). The amount of dietary EPA and DHA was used to predict adult butterfly mass, as well as the amount of EPA and DHA in the whole butterfly using linear regression. A Pearson correlation test was conducted to determine if there was a relationship between dietary treatment and the incidence of wing deformities.

Treatment effect was considered significant when p < 0.05, and, where significant differences occurred, treatment means were differentiated using the Tukey HSD multiple comparison test. The normality, homogeneity and independence of residuals were considered to evaluate the data and appropriateness of the statistical model used.

## Results

### Diet composition

Diets contained between 8.1–8.9% total lipid (Table 1). The fatty acid composition of the experimental diets was almost identical; with the exception of EPA and DHA and total n-3 FA, which reflected the experimental formulations (Table 1). More complete FA profiles are presented on a quantitative (μg FA ∙ mg^-1^ diet dry weight; S1 Table) and proportional basis (% contribution of individual FA weight out of total quantified FA weight; S2 Table).

**Table 1:** Total lipid (% dry weight) and fatty acid composition (μg FA mg^-1^ dry weight) of experimental diets^1^,^2^ fed to cabbage white butterfly larvae under laboratory conditions.

	Control	Lowest	Low	Medium	High
Total lipid	8.1	8.7	8.6	8.9	8.9
18:2n-6 (LNA)	9.9	11.2	10.7	11.8	12.8
18:3n-3 (ALA)	1.7	1.7	1.7	1.7	1.8
20:5n-3 (EPA)	0	0.4	0.8	1.1	1.6
22:6n-3 (DHA)	0	0.3	0.6	0.7	0.8
∑n-3	1.7	2.4	3.0	3.5	3.6
∑n-6	10.1	11.1	10.8	11.0	13.0

^1^ Values are means of three analytical replicates.

^2^ Ratio of EPA + DHA to canola oil in experimental diets: Control (0:1), Lowest (0.25:0.75), Low (0.5:0.5), Medium (0.75:0.25), High (1:0).

### Growth, development time and morphology

The number of days between hatch and pupation, between hatch and emergence, and between pupation and emergence were not different among treatments (Table 2). Larval (just prior to pupation) and pupal weights were not different among treatments (Table 3). The mortality rate varied among treatments, but did not increase as a function of dietary EPA + DHA inclusion (Table 2). Food consumption was initially recorded at 18 days post-hatch. However, many larvae pupated during the first week of food consumption recording. Consequently, food consumption was only recorded for 4 days in order to achieve minimum replication among blocks and treatments. However, mean daily food consumption was not significantly different among treatments during that time period (F _4, 18_ = 2.88; p = 0.062). Adult weight increased in a linear fashion with EPA + DHA inclusion, whereby adults reared on the high EPA + DHA treatment were 20% heavier than those reared on the control treatment. Statistically, adults reared on the high EPA + DHA treatment were significantly heavier than those reared on low, lowest, and control treatments; and butterflies in the medium and low EPA + DHA treatment were heavier than those in the lowest and control EPA + DHA treatments (Table 3).

**Table 2:** The number of days from hatch to pupation and emergence, and from pupation to emergence (mean ± standard deviation) ^1^, in *Pieris rapae* larvae fed experimental diets containing EPA and DHA.

	Control	Lowest	Low	Medium	High	F-stat	df	p-value^2^
Days to pupation	16.0 ± 1.5	15.3 ± 1.5	15.6 ± 1.1	16.0 ± 1.6	16.0 ± 1.4	1.86	4, 119	0.122
Days to emergence	24.3 ± 2.2	23.5 ± 2.0	23.6 ± 1.2	23.8 ± 1.6	24.3 ± 2.0	1.10	4, 108	0.362
Pupation to emergence	8.25 ± 0.9	8.17 ± 0.8	7.96 ± 0.5	8.10 ± 1.6	8.12 ± 1.3	0.35	4, 108	0.845
Number of mortalities	7	6	5	10	5	-		-

^1^ n for each measurement varied among treatments due to mortalities.

^2^Treatment p-value in randomized block design; block effect not significant for any measurement (p > 0.05).

**Table 3:** Larval, pupal, and butterfly weights (wet weight, mg), and morphological measurements^1^,^2^ (cm) in *Pieris rapae* larvae fed experimental diets containing EPA and DHA.

	Control	Lowest	Low	Medium	High	F-stat	df	p-value
Larvae weight	202.3 ± 32	217.9 ± 32	202.0 ± 48	206.5 ± 52	206.7 ± 25	0.50	4, 119	0.695
Pupae weight	184.3 ± 28	189.9 ± 20	192.3 ± 23	196.1 ± 18	186.3 ± 24	1.00	4, 119	0.411
Adult weight	105.6 ± 27^a^	106.4 ± 29^a^	116.1 ± 32^b^	120.6 ± 32^bc^	125.1 ± 34^c^	6.78	4, 108	**0.011**
Forewing span	4.57 ± 0.6^ab^	4.73 ± 0.5^a^	4.75 ± 0.4^a^	4.51 ± 0.5^ab^	3.94 ± 1.0^b^	4.48	4, 56	**0.003**
Hindwing span	3.18 ± 0.5^a^	3.13 ± 0.4^a^	3.20 ± 0.3^a^	2.85 ± 1.0^ab^	2.08 ± 0.3^b^	4.88	4, 56	**0.003**
Total wing length	2.59 ± 0.4^a^	2.33 ± 0.4^a^	2.40 ± 0.4^a^	2.27 ± 0.6^a^	1.58 ± 0.5^b^	6.04	4, 68	**0.001**
Forewing length	2.27 ± 0.3^a^	2.36 ± 0.3^a^	2.32 ± 0.2^ab^	2.23 ± 0.2^ab^	1.94 ± 0.4^b^	3.03	4, 68	**0.023**
Hindwing length	1.79 ± 0.2^a^	1.81 ± 0.1^a^	1.91 ± 0.3^a^	1.64 ± 0.3^ab^	1.36 ± 0.4^b^	3.51	4, 88	**0.012**
Wing span: weight	45.4 ± 15^ab^	54.7 ± 9.7^a^	42.9 ± 9.1^ab^	39.2 ± 9.6^b^	41.5 ± 12.1^ab^	3.46	4, 58	**0.014**

^1^Morphometric measurements were not possible to conduct on all highly deformed individuals; therefore n for each measurement varied among treatments.

^2^Different superscripts indicate significant difference among treatments.

**Fig 1:**
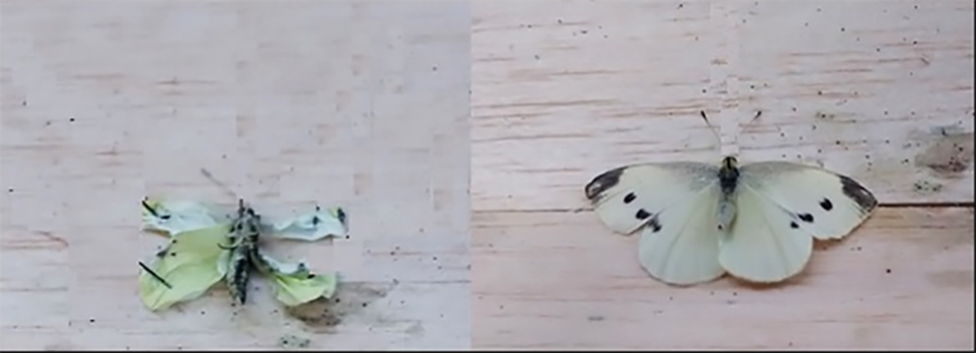
Example of cabbage butterflies (pinned to insect boards) fed experimental diets 48 hours after emergence: butterfly with deformed wings (left panel, 100% EPA + DHA diet) compared to a butterfly with intact wings (right panel, control diet).

**Fig 2:**
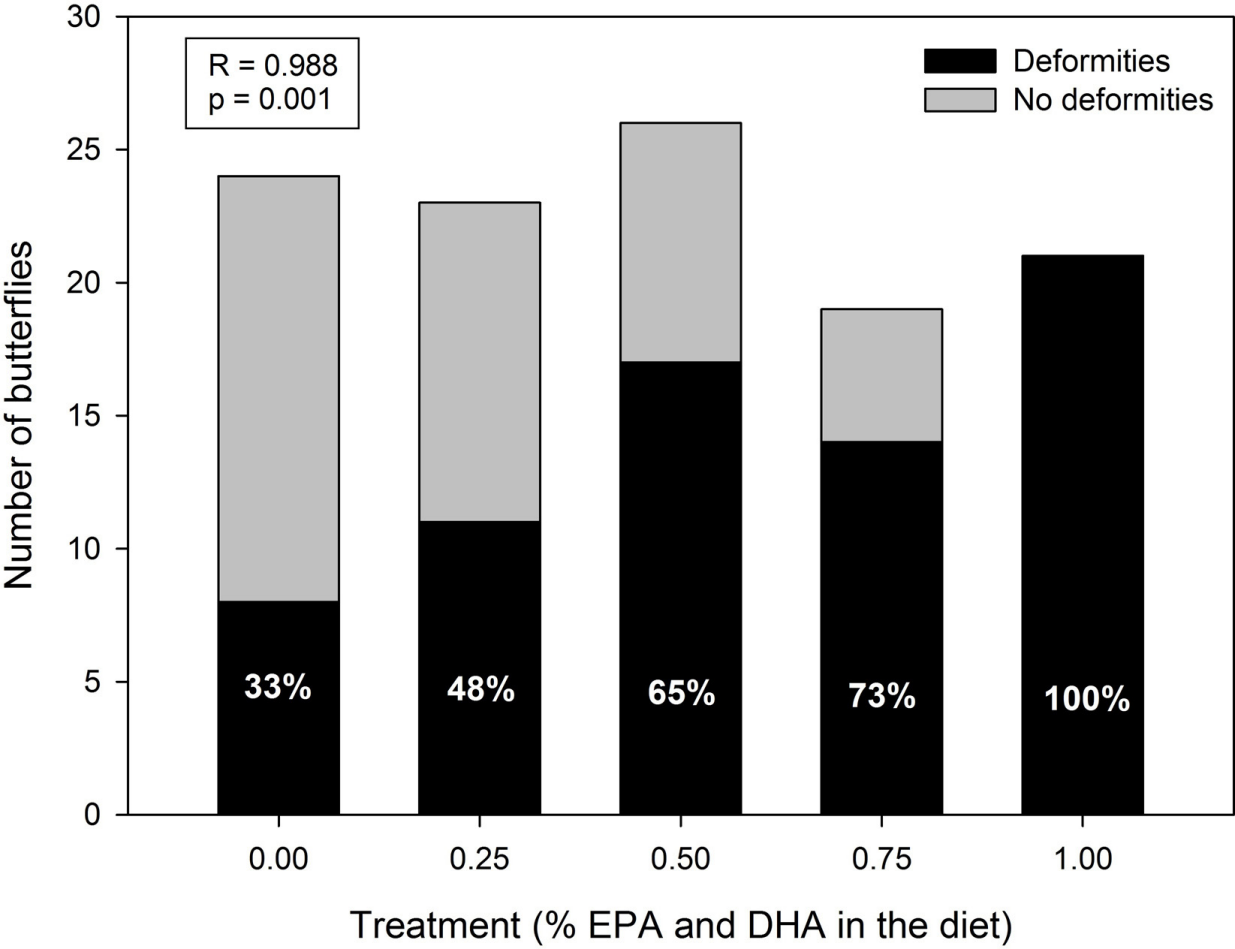
Incidence of wing deformities in Pieris rapae butterflies fed EPA and DHA enhanced diets in larval stage. The incidence of wing deformities correlates significantly with the level of EPA and DHA in the diet (correlation coefficient R = 0.988; p = 0.001).

Wing deformities were observed in a number of butterflies. Wings were considered deformed if they were significantly smaller and/or were wilted, folded, underdeveloped and/or non-functioning 48 h post emergence (Fig 1). Some individuals were highly deformed and attempts to measure wing morphology resulted in breakage of the wings, therefore those individuals could not be measured. Forewing span was significantly shorter in the high EPA + DHA dietary treatment (by at least 20%; Table 3). Hindwing span was ~50% shorter in the high EPA + DHA dietary treatment in comparison with the control, lowest and low EPA + DHA dietary treatments. Total wing length was shorter (by at least 40%) in adults reared on the high EPA + DHA dietary treatment compared with all other treatments. Forewing length was shorter in adults reared on the high EPA + DHA dietary treatment than the lowest and control treatments, by at least 15%. Hindwing length was shorter (by at least 30%) in adults reared on the high EPA + DHA dietary treatment than the lowest and low EPA + DHA dietary treatments, as well as the control. The ratio between wing span to total butterfly weight was higher in adults reared on the lowest EPA + DHA treatment compared with the medium treatment. Although butterflies in the control treatment also had wilted, folded, underdeveloped and/or non-functioning wing deformities (33% of individuals; see Discussion), there was nonetheless a strong positive correlation between the proportion of these type of deformities and dietary EPA and DHA level (correlation coefficient R = 0.99; p = 0.001) with the highest EPA and DHA diet resulting in 100% wing deformities (Fig 2).

### Whole butterfly total lipid and fatty acids

**Fig 3:**
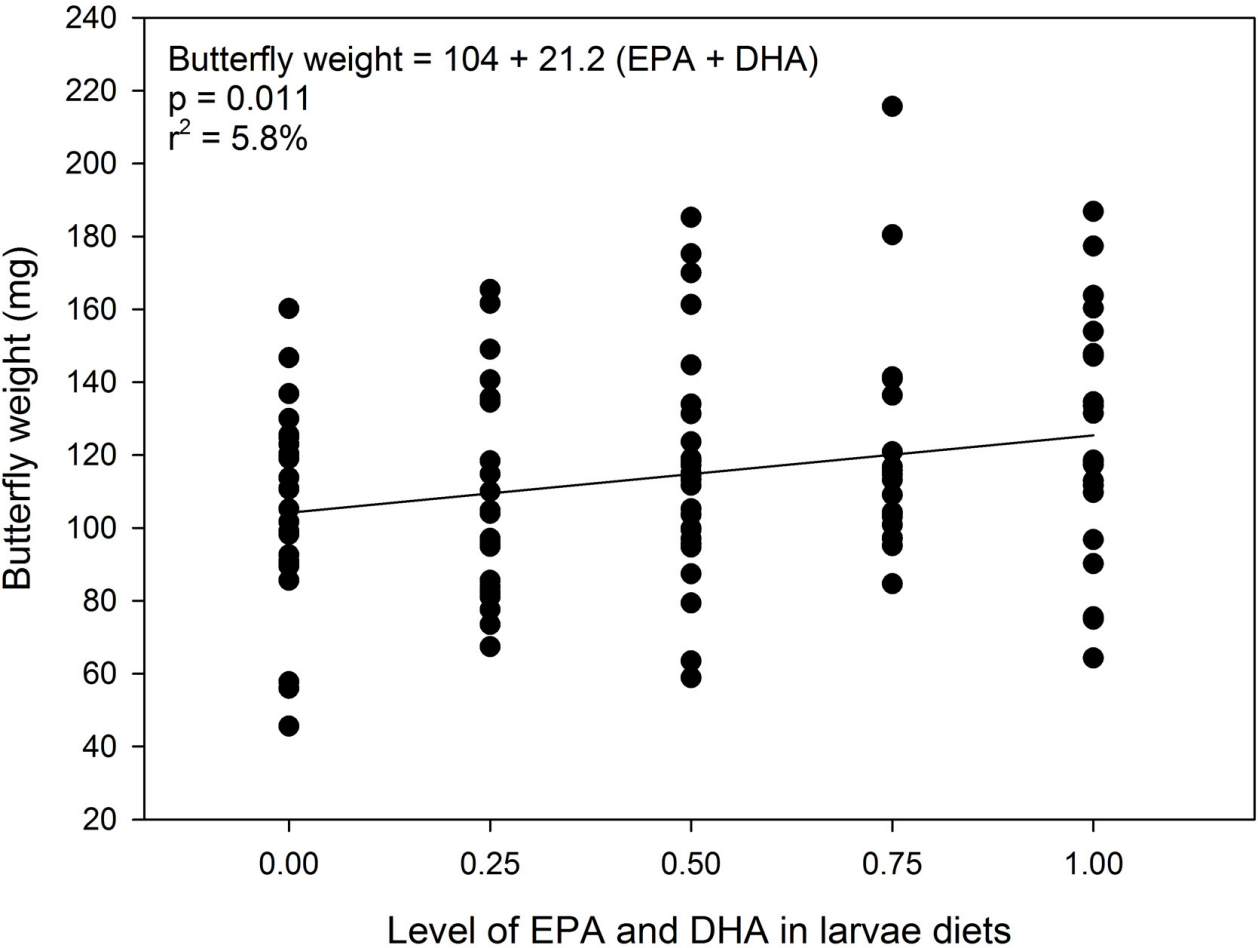
*Pieris rapae* butterfly weight (mg) with increasing levels of EPA and DHA in larval diets.

**Fig 4:**
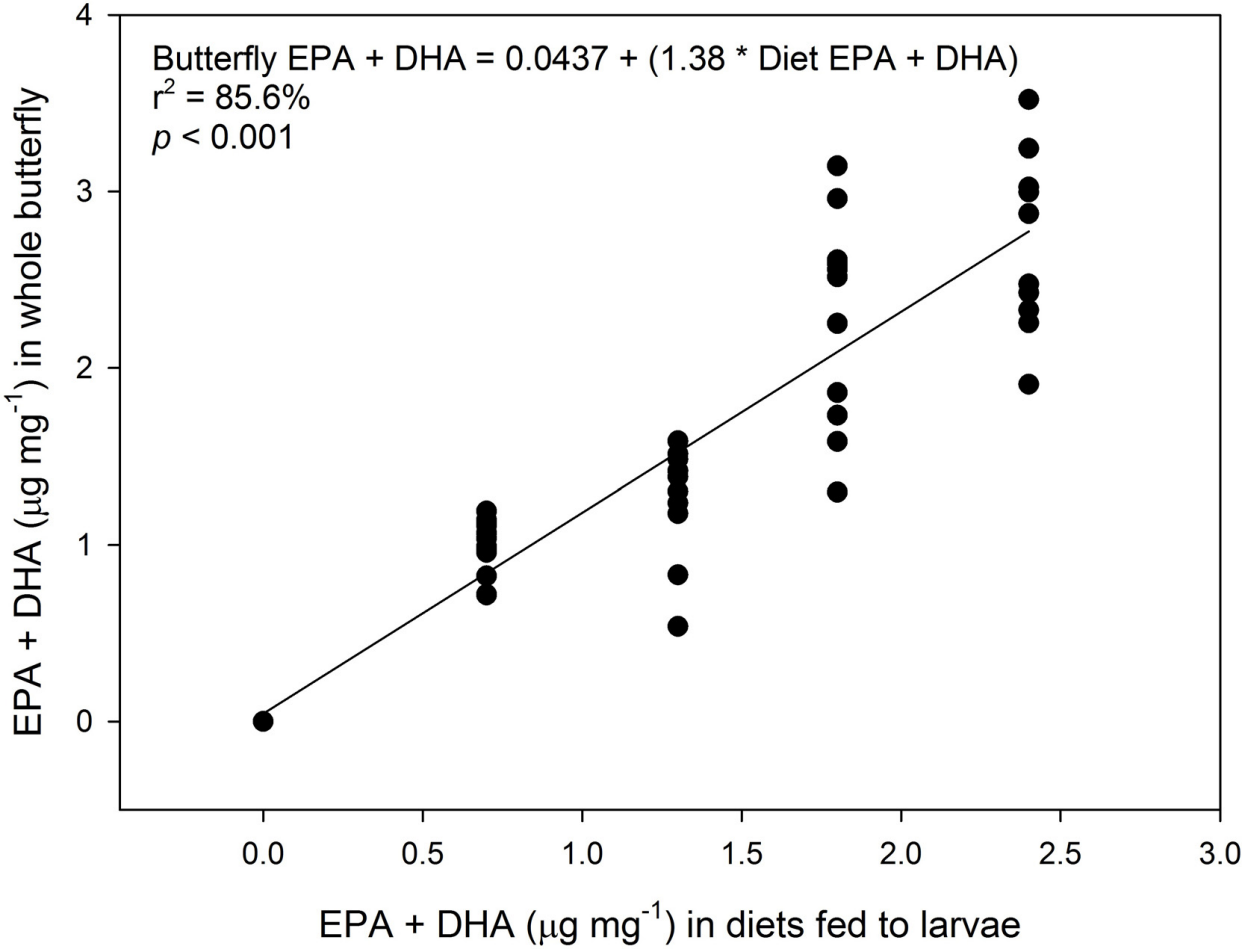
Linear relationship between dietary EPA + DHA and tissue EPA + DHA in *Pieris rapae* butterflies fed experimental diets.

Adults reared on the low and medium EPA + DHA diets had higher total body lipid than those reared on the control and lowest diets, while adults fed the high diet did not to those in other treatments (Table 4). There were differences in the amount of EPA and DHA (and their sum) stored in adults according to treatment, with EPA and DHA amounts highest in those reared on the medium and high EPA + DHA diets. Adults reared on the control diet did not contain EPA and DHA. Total n-3 FA content increased in adults with higher inclusion of dietary EPA and DHA in larval diets. The amounts of ALA and LNA found in the remaining lipid portion of the diet (from canola oil and wheat germ) were not different among treatments. Finally, adult weight (Fig 3) and the amount of EPA and DHA in the whole butterfly (Fig 4) increased with increasing amounts of dietary EPA and DHA. More complete FA profiles are presented on a quantitative (μg FA ∙ mg^-1^ whole butterfly dry weight; S3 Table) and proportional basis (% contribution of individual FA weight out of total quantified FA weight; S4 Table).

**Table 4:** Total lipid (% dry weight) and fatty acid composition (μg FA mg^-1^ dry weight) of whole *Pieris rapae* butterflies (n = 12 per treatment) that were fed experimental diets during their larval stage^1^.

	Control	Lowest	Low	Medium	High	F-stat _(4, 51)_	p-value
Total lipid	14.2 ± 2.6^a^	14.0 ± 2.1^a^	19.6 ± 1.8^b^	19.6 ± 2.6^b^	17.4 ± 3.1^ab^	4.91	**0.002**
18:2n-6 (LNA)	20.7 ± 4.5	19.9 ± 4.2	19.3 ± 4.2	20.2 ± 4.4	20.7 ± 4.5	1.32	0.275
18:3n-3 (ALA)	2.9 ± 0.5	2.8 ± 0.3	2.5 ± 0.4	2.9 ± 0.6	2.7 ± 0.4	1.46	0.228
20:5n-3 (EPA)	0.0 ± 0.0^a^	0.8 ± 0.1^b^	1.1 ± 0.2^b^	1.8 ± 0.1^c^	2.1 ± 0.4^c^	92.1	**< 0.001**
22:6n-3 (DHA)	0.0 ± 0.0^a^	0.1 ± 0.1^b^	0.2 ± 0.1^b^	0.4 ± 0.1^c^	0.6 ± 0.1^d^	81.0	**< 0.001**
∑ n-3	2.9 ± 0.4^a^	3.8 ± 0.4^b^	3.8 ± 0.7^b^	5.2 ± 1.2^c^	5.4 ± 0.9^c^	20.5	**0.001**
∑ n-6	20.8 ± 4.4	19.9 ± 4.2	19.4 ± 4.2	23.5 ± 6.5	21.0 ± 4.5	1.47	0.225

^1^Different superscripts indicate significant difference among treatments.

## Discussion

The n-3 LC-PUFA, EPA and DHA, are found in the highest concentrations in aquatic environments, especially at the level of primary producers and primarly consumers [11, 12]. Based on what is known concerning the positive bioactive effects of EPA and DHA on growth, development, and reproduction in aquatic invertebrates [3], we hypothesized that novel introduction of these FA to the terrestrial environment, via genetically engineered oilseeds, would influence terrestrial insects. We found that increasing the amount of EPA and DHA in larval diets resulted in a corresponding increase in the concentrations of these two FA in adult cabbage white butterflies upon emergence and directly correlated with changes in adult weight, and wing morphology.

The artificial diets contained the essential PUFA for this species (ALA and LNA), but also included increasing amounts of aquatic-sourced EPA and DHA, from 0 in the control treatment up to 2.4 μg ∙ mg^-1^ dry weight (or 9.1%) in the high EPA + DHA treatment. In order to put this into context, we estimated the amount of EPA and DHA found in a genetically engineered camelina seed with what might be found in leaf tissue based on the following approximate calculation. We point out that concentrations of EPA and DHA in vegetative tissues of genetically engineered oilseed crops have not yet been reported. One gram of camelina seed is expected to yield ~400 mg of oil, dry weight [14], which we estimated would include 44 and 28 μg mg^-1^ of EPA and DHA, respectively, based on proportions of these FA in the genetically engineered oil [16]. This amount is 30X higher than the amount of EPA and DHA provided in our highest EPA + DHA diet. Therefore, the EPA and DHA levels that we used in our diets were rather conservative compared with the estimated amount in genetically engineered seed. While some *Brassica* crop pest species (e.g. cabbage seedpod weevil, *Ceutorhynchus obstrictis*) do specialize in seedpod consumption in the larval stage, there are also some lepidopterans (e.g. diamondback moth, *Plutella xylostella;* alfalfa looper, *Autographica california*; and beet webworm, *Loxostege sticticalis*) that consume the seedpod and leaf tissue indiscriminately [27]. The lipid content of vegetative tissues is typically very low in the majority of plant species [13], for example, vegetative tissue from *C*. *sativa* is ~2% total lipid, dry weight [28]; therefore, when the same calculation is applied as above, the total amount of EPA + DHA is 3.6 μg mg^-1^ leaf tissue. This amount is still greater than that in our high diet treatment (2.4 μg mg^-1^ EPA + DHA). Therefore, the highest amounts of EPA and DHA included in the artificial diets in this study are close to, but slight underestimates of, those in leaves that would be consumed by leaf specialists (such as *P*. *rapae*) in genetically engineered oilseed crop fields. It should be noted that EPA and DHA biosynthesis may be controlled by seed-specific promoters (e.g., 15, 16), and therefore the transgene may be expressed in the seed only; however, the absence of EPA and DHA in other plant tissues has yet to be confirmed.

Dietary amounts of total lipid, ALA, and LNA, were relatively consistent across all treatments; the only FA that varied were EPA and DHA. Therefore, any treatment effects that we observed could be ascribed to EPA and DHA additions, rather than to changes in ALA and/or LNA. We found that inclusion of EPA and DHA in larval diets did not affect development time (days from hatch to pupation and emergence, and days from pupation to emergence) or the mortality rate in larvae. Also, larval and pupal masses were not different among treatments. Therefore, at least in the larval stage, these results indicate that dietary inclusion of EPA and DHA did not affect gross growth and development times, as is generally the case in aquatic invertebrates. Adult mass, however, varied as a function of dietary EPA and DHA inclusion, which suggests that tissue mass was more efficiently retained during metamorphosis in those individuals reared on EPA and DHA-containing diets. In contrast, those reared on the control and lowest diets presumably lost more weight during metamorphosis, as larval and pupal weights did not differ among treatments. Interestingly, while total body lipid in adults seemed to increase with dietary provision of EPA and DHA, total lipid was increasing up to a certain point after which, despite the addition of more EPA and DHA, it did not increase further (statistically). This suggests that the highest amounts of EPA and DHA may have been somewhat toxic to adults during the first 48 h post-metamorphosis, resulting in net metabolic costs and reduced body fat. However, our short term experiment did not address potential effects of dietary EPA and DHA on other key physiological endpoints in adults, including life span, mobility, sensory abilities, and overwintering and reproductive success; factors known to various affect the instrinsic rate of increase (r) of pest populations.

We found that increasing amounts of EPA and DHA in larval diets correlated strongly with deformities in butterfly wings. For example, forewing span and hindwing span were ~20 and 50% shorter, respectively, in adults reared on the high EPA + DHA treatment compared with the control. A previous study found that an induced dietary deficiency of ALA in the cabbage looper (*Trichoplusia ni* Hübner; Lepidoptera: Noctuidae) led to wing deformities when larvae of these moths were raised under increased temperature (up to 30°C) and humidity conditions (up to 95%) [29]. In other moth species ALA deficiency resulted in failure of normal adult emergence and wing development [30, 31]; therefore, it has been recommended that diets of lepidopterans include a source of ALA, such as linseed oil, to prevent wing deformities [32]. Further, ALA deficiency in Lepidoptera has been characterized by a failure of the adult to form properly during metamorphosis, with seemingly no other measured effects during larval development [33]. In our study, the amount of ALA was consistent across all treatments. Therefore, the observed effects were not due to differences in dietary ALA provision, but were more specifically related to the inclusion of EPA and DHA in larval diets. This evidence suggests there may be relationships between n-3 PUFA and wing morphology. Canavoso et al. [2] concluded that the function of n-3 PUFA in metamorphosis has yet to be clearly established.

There also appears to be a positive relationship between high humidity during the pupal stage and incidence of wing deformities [29]. Relative humidity in our study was unintentionally high, due to ambient conditions in the rearing containers at time of pupation and emergence, and this may have led to a greater incidence of deformities across all treatments, including the control. Nevertheless, we note that humidity was constant and equal among treatments and that the incidence of deformities nonetheless increased in a dose-response manner in the experimental treatments as a function of dietary EPA + DHA provision relative to the controls.

There was a positive linear relationship (r^2^ = 0.856) between dietary EPA and DHA provision and adult whole body levels of these two n-3 LC-PUFA confirming tissue incorporation of these FA in direct relation to dietary provision. The tissue incorporation of these FA in relation to diet is expected, as many studies have shown that FA composition of insects changes in response to changing levels of dietary PUFA [1], including in *Pieris brassicae* L.; Lepidoptera: Pieridae [34], a close relative of *P*. *rapae*. Our study is not the first to detect trace amounts of n-3 LC-PUFA in *Pieris* spp. However, LC-PUFA are only quantifiable in specific lipid classes of certain tissues [1]. For example, EPA was detected in *P*. *brassicae* in the phospholipid fractions of reproductive tissue and adult flight muscles, without EPA in the diet [34]. The detection of EPA in *P*. *brassicae* suggests that it was synthesized from ALA, as larvae were fed *Brassica oleracea* [35], which is not known to contain EPA, and further, it has been established that EPA can be synthesized from dietary ALA in other species of Lepidoptera [36]. *Pieris rapae* has a dietary requirement for ALA (the metabolic precursor to EPA), some of which can be converted to EPA [35]. Moreover, ALA and 20-carbon LC-PUFA are known to be associated with flight activity in insects [36]. These studies suggest that EPA may be an important FA with respect to some physiological functions of *Pieris* spp.

Biosynthesis and tissue-specific incorporation of certain FA are tightly regulated processes [1]. Clearly, if EPA is synthesized and actively incorporated in particular tissues (e.g. reproductive and/or locomotive), then it is highly likely that it is required for the optimum functionality of that tissue. However, in our study, dietary provision of EPA (and DHA) appeared to have been more detrimental than beneficial at levels expected to be found in genetically engineered oilseeds. Our results confirm previous work that suggests the n-3 PUFA (at least ALA and EPA) appear to be relevant in adult metamorphosis and wing development; however, the exact mechanism and relationship of each individual n-3 PUFA to these processes is unknown [2].

EPA also plays a role in immune function in insects [18]. EPA and ARA are precursors to eicosanoids, which are highly bioactive compounds that function in immunity, mediating and co-ordinating cellular and humoral responses at specific and crucial moments; e.g., as immediate reactions to infection [18]. The eicosanoids derived from EPA have different functions than those derived from ARA; a skew in the ratio between these FA may have consequences on immune function [18]. It is possible that high amounts of EPA in the tissue led to a high EPA/ARA ratio which potentially may have impaired immune function at a critical time, coupled with high humidity conditions (which may occur naturally in crop fields depending on the vagaries of local climate conditions) that are known to result in higher frequencies of wing deformities [29].

Our preliminary results based on the supplementation of EPA and DHA in artificial rearing diets for *P*. *rapae* larvae suggest that these compounds can influence wing development; however, we stress that further testing is required to more fully understand the impacts of these compounds on herbivorous insects including crop pests, and on the consumers of these insects. There are challenges associated with using experimental artificial diets as a model and extrapolating results from these conditions to nature, although the line of *P*. *rapae* used in our experiment were likely highly adapted to artificial diets through selection [19, 37]. However, the next step in risk assessment should involve using the actual plants (both in laboratory and in fully contained field trials) engineered to produce EPA and DHA to validate our findings. Based on previous studies with other lepidopterans, it is clear that EPA is synthesized in certain tissues for specific functions. Furthermore, n-3 PUFA seem to be generally involved in lepidopteran metamorphosis and emergence. However, we do not know the full scope of potential effects that access to dietary EPA and DHA during the larval period might have on post-emerged adults; for example, how these FA may affect adult lifespan, flying ability, sensory ability, and/or overwintering and reproductive success. Our study illuminates some of the possible implications surrounding the impending regulation of genetically engineered oilseeds that endogenously produce EPA and DHA. We suggest that, in order to make informed decisions with respect to agricultural and environmental policies and management of genetically engineered oilseeds (e.g. see 38), studies similar to ours (as well as contained field studies) need to be conducted in order to understand more completely the potential global environmental impacts of widespread commercial production of these novel crops on agroecosystems.

## Supporting Information

S1 Table Total lipid (% dry weight) and FA composition (μg ∙ mg^-1^) of diets fed to the larval cabbage butterfly (n = 3) (DOCX)

S2 Table Total lipid (%) and FA composition (% total FA) of diets fed to the larval cabbage butterfly (n = 12) (DOCX)

S3 Table Total lipid (% dry weight) and FA composition (μg ∙ mg-1) of whole butterflies (n = 12) that were fed experimental diets during their larval stage (DOCX)

S4 Table Total lipid (%) and FA composition (% total FA) of whole butterflies that were fed experimental diets during their larval stage (DOCX)

## References

[1] Stanley-Samuelson DW, Jurenka RA, Cripps CC, Blomquist GL, de Renobales M. Fatty acids in insects: Composition, metabolism, and biological significance. Arch Insect Biochem. 1988; 9: l–33.

[2] Canavoso LE, Jouni ZE, Karnas KJ, Pennington JE, Wells MA. Fat metabolism in insects. Ann Rev Nutr. 2001; 21: 23–46. 11375428 10.1146/annurev.nutr.21.1.23

[3] Arts MT, Brett MT, Kainz MJ. Lipids in Aquatic Ecosystems. Springer, New York; 2009.

[4] Parrish C. Essential fatty acids in aquatic food webs In: Arts MT, Brett MT, Kainz MJ, editors. Lipids in Aquatic Ecosystems. Springer: New York; 2009 pp. 309–326

[5] Kainz MJ, Arts MT, Mazumber A. Essential fatty acids in the planktonic food web and their ecological role for higher trophic levels. Limnol Oceanogr. 2004; 49: 1784–1793.

[6] Hixson SM, Sharma B, Kainz MJ, Wacker A, Arts MT. Production, distribution, and abundance of long-chain omega-3 polyunsaturated fatty acids: A fundamental dichotomy between freshwater and terrestrial ecosystems. Environ Rev. 2015; 10.1139/er-2015-0029

[7] Twining CW, Brenna JT, Hairston NG, Flecker AS. Highly unsaturated fatty acids in nature: what we know and what we need to learn. Oikos. 2015; 10.1111/oik.02910

[8] FAO. 2014. The State of World Fisheries and Aquaculture, Food and Agriculture Organization of the United Nations Rome Italy; 2014 pp. 3–93.

[9] Tacon AG, Metian M. Feed matters: Satisfying the feed demand of aquaculture. Rev Fish Sci Aquacult. 2015; 23: 1–10.

[10] Betancor M, Sprague M, Usher S, Sayanova O, Campbell PJ, Napier JA, et al A nutritionally-enhanced oil from transgenic *Camelina sativa* effectively replaces fish oil as a source of eicosapentaenoic acid for fish. Sci Rep. 2015; 5: 8104 10.1038/srep08104 25632018 PMC4309969

[11] Hixson SM. Fish nutrition and current issues in aquaculture: The balance in providing safe and nutritious seafood, in an environmentally sustainable manner. J Aquac Res Develop. 2014; 5: 234.

[12] Kitessa S, Abeywardena M, Wijesundera C, Nichols PD. DHA-containing oilseed: A timely solution for the sustainability issues surrounding fish oil sources of the health-benefitting long-chain omega-3 oils. Nutrients. 2014; 6: 2035–2058. 10.3390/nu6052035 24858407 PMC4042577

[13] Napier JA, Usher S, Haslam RP, Ruiz-Lopez N, Sayanova O. Transgenic plants as a sustainable, terrestrial source of fish oil. Eur J Lipid Sci Technol. 2015; 117: 1317–1324. 26900346 10.1002/ejlt.201400452 PMC4744972

[14] Mansour MP, Shrestha P, Belide S, Petrie JR, Nichols PD, Singh SP. Characterization of oilseed lipids from DHA-producing *Camelina sativa*: A new transformed land plant containing long-chain omega-3 oils. Nutrients. 2014; 6: 776–789. 10.3390/nu6020776 24566436 PMC3942731

[15] Petrie JR, Shrestha P, Belide S, Kennedy Y, Lester G, Liu Q, et al Metabolic engineering *Camelina sativa* with fish oil-like levels of DHA. PLoS One. 2014; 9: e85061 10.1371/journal.pone.0085061 24465476 PMC3897407

[16] Ruiz-Lopez N, Haslam R, Napier J, Sayanova O. Successful high-level accumulation of fish oil omega-3 long-chain polyunsaturated fatty acids in a transgenic oilseed crop. The Plant J. 2014; 77: 198–208. 10.1111/tpj.12378 24308505 PMC4253037

[17] Stanley DW, Miller JS. Eicosanoid actions in insect cellular immune functions. Entomol Exp Appl. 2006; 119: 1–13.

[18] Stanley D, Kim Y. Eicosanoid signaling in insects: from discovery to plant protection. Crit Rev Plant Sci. 2014; 33: 20–63.

[19] Rotem K, Agrawal AA, Laima K. Parental effects in *Pieris rapae* in response to variation in food quality: adaptive plasticity across generations? Ecol Entomol. 2003; 28: 211–218.

[20] Root RB, Kareiva PM. The search for resources by cabbage butterflies (*Pieris rapae*): Ecological consequences and adaptive significance of Markovian movements in a patchy environment. Ecology. 1984; 65: 147–165.

[21] Myers JH. Effect of physiological condition of the host plant on the ovipositional choice of the cabbage white butterfly, *Pieris rapae*. J Anim Ecol. 1985; 54: 193–204.

[22] Webb SE, Shelton AM. Laboratory rearing of the imported cabbageworm New York State Agricultural Experiment Station Bulletin, Cornell University, Ithaca NY. 1988; 122: 1–6 (ISSN 0362-0069).

[23] Chai S, Srygley RB. Predation and the flight, morphology, and temperature of neotropical rain-forest butterflies. The Amer Nat. 1990; 135: 748–765.

[24] Folch J, Lees M, Sloane-Stanley GH. A simple method for the isolation and purification of total lipids from animal tissues. J Biol Chem. 1957; 226: 497–509. 13428781

[25] McMeans BC, Arts MT, Rush SA, Fisk AT. Seasonal patterns in fatty acids and stable isotopes of *Calanus hyperboreus* (Copepoda, Calanoida) from Cumberland Sound, Baffin Island. Mar Biol. 2012; 159: 1095–1105.

[26] Christie WW. Preparation of derivates of fatty acids In: Christie WW, editor. Lipid Analysis: Isolation, Separation and Structural Analysis of Lipids. 3rd ed. J. Barnes and Associates; 2003 pp. 205–225.

[27] Canola Council of Canada. Chapter 10 Insects Canola Grower’s Manual. Canola Council of Canada Publication. Agriculture and Agri-Food Canada; 2014.

[28] Peiretti PG, Meineri G. Fatty acids, chemical composition and organic matter digestibility of seeds and vegetative parts of false flax (*Camelina sativa* L.) after different lengths of growth. Anim Feed Sci Tech. 2007; 133: 341–350.

[29] Grau PA, Terriere LC. Fatty acid profile of the cabbage looper, *Trichoplusia ni*, and the effect of diet and rearing conditions. J Insect Physiol. 1971; 17: 1637–1649.

[30] Dadd RH. Long-chain polyenoics and the essential dietary fatty acid requirement of the waxmoth, *Galleria mellonella*. J Insect Physiol. 1983; 29: 779–786.

[31] Stanley-Samuelson DW, Dadd RH. Polyunsaturated fatty acids in the lipids from adult *Galleria mellonella* reared on diets to which only one unsaturated fatty acid had been added. Insect Biochem. 1984; 3: 321–327.

[32] Morton AC. Rearing butterflies on artificial diets. 1979; 81 : 221–227.

[33] Dadd RH. Nutrition: organisms In:. Kerkurt GA, Gilbert LI, editors. Comprehensive Insect Physiology, Biochemistry and Pharmacology. Oxford, UK: Pergamon; 1985 pp. 313–390.

[34] Turunen S. Lipid utilization in adult *Pieris brassicae* with special reference to the role of linolenic acid. J Insect Physiol. 1974; 20: 1257 4853011 10.1016/0022-1910(74)90231-5

[35] Parnanen S, Turunen S. Eicosapentaenoic acid in tissue lipids of *Pieris brassicae*. Experientia. 1986; 43: 215–217.

[36] Stanley-Samuelson DW. Prostaglandins and related eicosanoids in insects In:. Evans PD, editor. Advances in insect physiology. Academic Press San Diego CA; 1994. pp. 115–212.

[37] Boller E. Behavioral aspects of mass-rearing of insects. Entomophaga. 1972 17: 9–25.

[38] Snow AA, Andow DA, Gepts P, Hallerman EM, Power A, Tiedje JM, et al Genetically engineered organisms and the environment: Current status and recommendations. Ecol Appl. 2004; 15: 377–404.

