# Supplementary material for: Long-Chain Omega-3 Polyunsaturated Fatty Acids Have Developmental Effects on the Crop Pest, the Cabbage White Butterfly Pieris rapae
Source: PLoS One. 2016 Mar 24;11(3):e0152264. doi: 10.1371/journal.pone.0152264 (PMC4806837; doi:10.1371/journal.pone.0152264)
Supplement: S1 Table — (DOCX) [file pone.0152264.s001.docx]

S1 Table. Total lipid (% dry weight) and FA composition (µg ∙ mg^-1^) of diets fed to the larval cabbage butterfly (n=3)

|  | **Control** | **Lowest** | **Low** | **Medium** | **High** |
| --- | --- | --- | --- | --- | --- |
| Total lipid | 8.1 | 8.7 | 8.6 | 8.9 | 8.9 |
| 14:0 | 1.2 | 0.8 | 1.2 | 1.0 | 1.0 |
| 16:0 | 3.6 | 3.5 | 3.4 | 3.7 | 4.0 |
| 18:0 | 1.2 | 0.8 | 1.2 | 1.0 | 1.0 |
| 18:1n-9 | 3.2 | 3.1 | 3.7 | 3.1 | 3.9 |
| 18:2n-6 | 9.9 | 11.2 | 10.7 | 11.8 | 12.8 |
| 18:3n-3 | 1.7 | 1.7 | 1.7 | 1.7 | 1.8 |
| 20:5n-3 | 0 | 0.4 | 0.8 | 1.1 | 1.6 |
| 22:6n-3 | 0 | 0.3 | 0.6 | 0.7 | 0.8 |
| ∑SFA | 5.7 | 5.2 | 6.0 | 5.7 | 6.3 |
| ∑MUFA | 4.5 | 4.4. | 4.4 | 4.5 | 4.6 |
| ∑PUFA | 11.8 | 13.6 | 13.9 | 13.6 | 16.6 |
| ∑n-3 | 1.7 | 2.4 | 3.0 | 3.5 | 3.6 |
| ∑n-6 | 10.1 | 11.1 | 10.8 | 11.0 | 13.0 |
