# Supplementary material for: Long-Chain Omega-3 Polyunsaturated Fatty Acids Have Developmental Effects on the Crop Pest, the Cabbage White Butterfly Pieris rapae
Source: PLoS One. 2016 Mar 24;11(3):e0152264. doi: 10.1371/journal.pone.0152264 (PMC4806837; doi:10.1371/journal.pone.0152264)
Supplement: S2 Table — (DOCX) [file pone.0152264.s002.docx]

S2 Table. Total lipid (%) and FA composition (% total FA) of diets fed to the larval cabbage butterfly (n=12)

|  | **Control** | **Lowest** | **Low** | **Medium** | **High** |
| --- | --- | --- | --- | --- | --- |
| Total lipid | 8.1 | 8.7 | 8.6 | 8.9 | 8.4 |
| 14:0 | 5.6 | 3.6 | 4.8 | 3.9 | 3.8 |
| 16:0 | 16.1 | 15.1 | 14.1 | 14.3 | 14.5 |
| 18:0 | 4.2 | 2.4 | 2.7 | 2.3 | 2.4 |
| 18:1n-9 | 14.8 | 14.5 | 12.8 | 11.6 | 10.5 |
| 18:2n-6 | 45.5 | 48.1 | 43.4 | 45.7 | 46.1 |
| 18:3n-3 | 7.8 | 7.2 | 7.1 | 6.7 | 6.7 |
| 20:5n-3 | 0 | 1.7 | 3.1 | 4.3 | 5.6 |
| 22:6n-3 | 0 | 1.3 | 2.2 | 2.8 | 3.5 |
| ∑SFA | 26.0 | 22.3 | 24.5 | 22.3 | 23.2 |
| ∑MUFA | 20.3 | 19.1 | 18.8 | 17.5 | 16.7 |
| ∑PUFA | 53.7 | 58.6 | 56.7 | 60.2 | 60.2 |
| ∑n-3 | 7.8 | 10.5 | 12.4 | 13.7 | 13.3 |
| ∑n-6 | 45.8 | 48.2 | 44.2 | 46.4 | 46.9 |
