# Supplementary material for: Long-Chain Omega-3 Polyunsaturated Fatty Acids Have Developmental Effects on the Crop Pest, the Cabbage White Butterfly Pieris rapae
Source: PLoS One. 2016 Mar 24;11(3):e0152264. doi: 10.1371/journal.pone.0152264 (PMC4806837; doi:10.1371/journal.pone.0152264)
Supplement: S3 Table — (DOCX) [file pone.0152264.s003.docx]

S3 Table. Total lipid (% dry weight) and FA composition (µg ∙ mg^-1^) of whole butterflies (n=12) that were fed experimental diets during their larval stage

|  | **Control** | **Lowest** | **Low** | **Medium** | **High** | **F-stat** | **p-value** |
| --- | --- | --- | --- | --- | --- | --- | --- |
| Total lipid | 14.2 ± 2.6a | 14.0 ± 2.1a | 19.6 ± 1.8b | 19.6 ± 2.6b | 17.4 ± 3.1ab | 4.91 | 0.002 |
| 14:0 | 3.7 ± 1.0a | 1.9 ± 0.8b | 0.8 ± 0.2c | 1.0 ± 0.1c | 1.1 ± 0.2c | 59.5 | 0.001 |
| 16:0 | 7.3 ± 1.7 | 7.2 ± 2.2 | 6.9 ± 1.9 | 8.8 ± 2.8 | 7.4 ± 1.9 | 1.48 | 0.223 |
| 18:0 | 2.9 ± 0.4ab | 2.7 ± 0.4ab | 2.3 ± 0.4a | 2.9 ± 0.8b | 2.6 ± 0.4ab | 2.72 | 0.040 |
| 18:1n-9 | 12.8 ± 3.0 | 13.7 ± 4.3 | 12.6 ± 3.6 | 14.6 ± 4.5 | 12.7 ± 3.5 | 0.67 | 0.613 |
| 18:2n-6 | 20.7 ± 4.5 | 19.9 ± 4.2 | 19.3 ± 4.2 | 20.2 ± 4.4 | 20.7 ± 4.5 | 1.32 | 0.275 |
| 18:3n-3 | 2.9 ± 0.5 | 2.8 ± 0.3 | 2.5 ± 0.4 | 2.9 ± 0.6 | 2.7 ± 0.4 | 1.46 | 0.228 |
| 20:5n-3 | 0.0 ± 0.0a | 0.8 ± 0.1b | 1.1 ± 0.2b | 1.8 ± 0.1c | 2.1 ± 0.4c | 92.1 | < 0.001 |
| 22:6n-3 | 0.0 ± 0.0a | 0.1 ± 0.1b | 0.2 ± 0.1b | 0.4 ± 0.1c | 0.6 ± 0.1d | 81.0 | < 0.001 |
| ∑SFA | 15.1 ± 2.5a | 13.1 ± 2.5ab | 10.9 ± 2.5b | 13.7 ± 3.4ab | 12.2 ± 2.4ab | 3.61 | 0.012 |
| ∑MUFA | 15.6 ± 3.2 | 16.1 ± 4.5 | 14.4 ± 4.0 | 16.7 ± 4.8 | 14.7 ± 3.5 | 0.73 | 0.578 |
| ∑PUFA | 23.6 ± 4.6 | 23.7 ± 4.6 | 23.2 ± 4.1 | 28.6 ± 7.1 | 26.5 ± 4.5 | 2.39 | 0.064 |
| ∑n-3 | 2.9 ± 0.4a | 3.8 ± 0.4b | 3.8 ± 0.7b | 5.2 ± 1.2c | 5.4 ± 0.9c | 20.5 | 0.001 |
| ∑n-6 | 20.8 ± 4.4 | 19.9 ± 4.2 | 19.4 ± 4.2 | 23.5 ± 6.5 | 21.0 ± 4.5 | 1.47 | 0.225 |
