# Supplementary material for: Long-Chain Omega-3 Polyunsaturated Fatty Acids Have Developmental Effects on the Crop Pest, the Cabbage White Butterfly Pieris rapae
Source: PLoS One. 2016 Mar 24;11(3):e0152264. doi: 10.1371/journal.pone.0152264 (PMC4806837; doi:10.1371/journal.pone.0152264)
Supplement: S4 Table — (DOCX) [file pone.0152264.s004.docx]

S4 Table. Total lipid (%) and FA composition (% total FA) of whole butterflies that were fed experimental diets during their larval stage

|  | Control | Lowest | Low | Medium | High |
| --- | --- | --- | --- | --- | --- |
| Total lipid | 14.2 ± 2.6 | 14.0 ± 2.1 | 19.6 ± 1.8 | 19.6 ± 2.6 | 17.4 ± 3.1 |
| 14:0 | 7.1 ± 2.4 | 3.7 ± 1.8 | 1.8 ± 0.4 | 1.8 ± 0.6 | 2.2 ± 0.5 |
| 16:0 | 13.3 ± 1.4 | 13.4 ± 1.4 | 14.2 ± 1.1 | 14.6 ± 1.0 | 13.7 ± 1.1 |
| 18:0 | 5.3 ± 0.5 | 5.3 ± 0.8 | 4.8 ± 0.6 | 5.0 ± 0.7 | 4.9 ± 0.8 |
| 18:1n-9 | 23.4 ±1.9 | 25.4 ± 2.8 | 25.9 ± 2.6 | 24.4 ± 1.6 | 23.5 ± 2.4 |
| 18:2n-6 | 37.9 ± 2.2 | 37.6 ± 1.5 | 40.0 ± 1.9 | 39.2 ± 1.5 | 38.7 ± 1.1 |
| 18:3n-3 | 5.3 ± 0.6 | 5.5 ± 0.7 | 5.2 ± 0.4 | 5.0 ± 0.4 | 5.2 ± 0.3 |
| 20:5n-3 | 0.0 ± 0.0 | 1.6 ± 0.3 | 2.3 ± 0.2 | 3.1 ± 0.1 | 3.9 ± 0.2 |
| 22:6n-3 | 0.0 ± 0.0 | 0.3 ± 0.2 | 0.4 ± 0.1 | 0.7 ± 0.1 | 1.2 ± 0.1 |
| ∑SFA | 28.0 ± 2.1 | 24.8 ± 2.1 | 22.6 ± 1.0 | 23.2 ± 1.4 | 22.9 ± 1.2 |
| ∑MUFA | 28.6 ± 1.3 | 30.1 ± 2.1 | 29.4 ± 2.7 | 28.2 ± 1.5 | 27.4 ± 2.2 |
| ∑PUFA | 43.3 ± 1.4 | 45.0 ± 2.0 | 47.9 ± 2.4 | 48.5 ± 1.7 | 49.6 ± 1.3 |
| ∑n-3 | 5.3 ± 0.6 | 7.4 ± 0.9 | 7.9 ± 0.6 | 8.8 ± 0.5 | 10.3 ± 0.5 |
| ∑n-6 | 37.9 ± 1.9 | 37.6 ± 1.5 | 40.1 ± 1.9 | 39.6 ± 1.5 | 39.2 ± 1.1 |
